# Magnesium intake, plasma C-peptide, and colorectal cancer incidence in US women: a 28-year follow-up study

**DOI:** 10.1038/bjc.2012.76

**Published:** 2012-03-13

**Authors:** X Zhang, E L Giovannucci, K Wu, S A Smith-Warner, C S Fuchs, M Pollak, W C Willett, J Ma

**Affiliations:** 1 Department of Medicine, Channing Laboratory, Brigham and Women's Hospital and Harvard Medical School, 401 Park Drive, Boston, MA 02115, USA; 2 Department of Nutrition, Harvard School of Public Health, Boston, MA, USA; 3 Department of Epidemiology, Harvard School of Public Health, Boston, MA, USA; 4 Department of Medical Oncology, Dana-Farber Cancer Institute and Harvard Medical School, Boston, MA, USA; 5 Department of Oncology, McGill University and Lady Davis Research Institute, 3999 Rue Côte Sainte Catherine, Montreal, Quebec, Canada H3T 1E2

**Keywords:** magnesium, calcium, insulin resistance, plasma C-peptide, colorectal cancer, incidence

## Abstract

**Background:**

Laboratory studies suggest a possible role of magnesium intake in colorectal carcinogenesis but epidemiological evidence is inconclusive.

**Method:**

We tested magnesium–colorectal cancer hypothesis in the Nurses’ Health Study, in which 85 924 women free of cancer in 1980 were followed until June 2008. Cox proportional hazards regression models were used to estimate multivariable relative risks (MV RRs, 95% confidence intervals).

**Results:**

In the age-adjusted model, magnesium intake was significantly inversely associated with colorectal cancer risk; the RRs from lowest to highest decile of total magnesium intake were 1.0 (ref), 0.93, 0.81, 0.72, 0.74, 0.77, 0.72, 0.75, 0.80, and 0.67 (*P*_trend_<0.001). However, in the MV model adjusted for known dietary and non-dietary risk factors for colorectal cancer, the association was significantly attenuated; the MV RRs were 1.0 (ref), 0.96, 0.85, 0.78, 0.82, 0.86, 0.84, 0.91, 1.02, and 0.93 (*P*_trend_=0.77). Similarly, magnesium intakes were significantly inversely associated with concentrations of plasma C-peptide in age-adjusted model (*P*_trend_=0.002) but not in multivariate-adjusted model (*P*_trend_=0.61). Results did not differ by subsite or modified by calcium intakes or body mass index.

**Conclusion:**

These prospective results do not support an independent association of magnesium intake with either colorectal cancer risk or plasma C-peptide levels in women.

Colorectal cancer remains the third most common type of cancer in both men and women in the United States and ∼49 380 Americans are estimated to die of colorectal cancer in 2011 (Siegel *et al*, 2011). Although dietary factors have been hypothesised to play an important role in colorectal carcinogenesis (World Cancer Research Fund, 2007), only a few dietary factors have been consistently identified to be associated with colorectal cancer risk.

Magnesium is an essential mineral needed for >300 biochemical reactions, and plays an important role in genetic stability, DNA synthesis, and glucose metabolism (Saris *et al*, 2000). Low blood levels of magnesium (hypomagnesemia) are often seen in individuals with type 2 diabetes, and magnesium might improve insulin sensitivity in healthy individuals (Paolisso *et al*, 1992) and in type 2 diabetics (Paolisso *et al*, 1989; Song *et al*, 2006). In addition, magnesium intake has been shown to decrease insulin levels (Ma *et al*, 1995; Fung *et al*, 2003; Song *et al*, 2004) and is associated with a lower risk of type 2 diabetes (Lopez-Ridaura *et al*, 2004; Song *et al*, 2004; Larsson and Wolk, 2007), a potential risk factor for colorectal cancer (Giovannucci *et al*, 2010). Multiple lines of evidence suggest a role of hyperinsulinemia and insulin resistance in colorectal cancer (Giovannucci, 2007; Pisani, 2008), implicating that factors such as magnesium that influence insulin levels might affect colorectal cancer risk.

Despite the potential mechanisms, evidence from observational studies has been inconclusive. Among the six large prospective cohort studies published to date, one study of Swedish women showed a significant inverse association between magnesium intake and colorectal cancer incidence (relative risk (RR)=0.59) (Larsson *et al*, 2005); three studies (Folsom and Hong, 2006; Lin *et al*, 2006; van den Brandt *et al*, 2007) showed non-significant inverse associations (RRs ranged between 0.80–0.91), one study showed a non-significant positive association (RR=1.27) (Li *et al*, 2011), and one study conducted in Japan showed a significant inverse association for men (RR=0.48) but not for women (RR=1.09) (Ma *et al*, 2010). In addition, with similar chemical properties, magnesium and calcium share the same homeostatic control system and may antagonise each other physiologically (Iseri and French, 1984). Studies showed that magnesium may compete with calcium for intestinal absorption and transport (Flatman, 1991). A low calcium:magnesium ratio in the lumen may activate the transport of magnesium, whereas a high calcium intake reduces absorption of both magnesium and calcium (Hardwick *et al*, 1991). Magnesium intake has also been associated with a lower risk of colorectal adenomas and hyperplastic polyps particularly among those with a low ratio of calcium-to-magnesium intake (Ca:Mg) in a case–control study (Dai *et al*, 2007), indicating a possible interaction between calcium and magnesium intakes in relation to colorectal cancer risk. Lack of consideration of this potential interaction may partly explain the inconsistency of previous findings.

The aim of this study is to examine the association between magnesium intake and colorectal cancer risk in the Nurses’ Health Study (NHS) and evaluate whether the association is modified by calcium intake. In addition, given that the potential effect of magnesium on colorectal cancer might be mediated through improving insulin sensitivity, we also tested whether plasma levels of C-peptide, a validated marker for insulin secretion (Bonser and Garcia-Webb, 1984), vary by magnesium intake.

## Materials and Methods

### Study population

Detailed information on the NHS has been described elsewhere (Willett, 1998). In brief, the NHS is an ongoing cohort study established in 1976 including 121 700 married female registered nurses at baseline who were 30–55 years old and resided in 11 states in the United States. Since 1976, biennial questionnaires were completed by participants to collect information on demographics, lifestyle factors, medical history, and disease outcomes. The follow-up rate has been >90%. In this analysis, we treated 1980 as the baseline, because that is when the magnesium intake was first assessed. We excluded participants with a history of cancer (except for non-melanoma skin cancer), with ulcerative colitis, with extreme baseline total energy intake (<600 or >3500 kcal per day) or with missing or extreme magnesium intakes (i.e., beyond 3 s.d. for log-transformed magnesium intake), which left 85 924 women for the analysis. This study has been approved by the institutional review board at the Brigham and Women's Hospital (Boston, MA, USA).

### Identification of incident colorectal cancer cases

Participants reported cancer and other disease outcomes in biennial questionnaires. With permission from the study participants, researchers obtained their medical records and pathological reports and, while blinded to exposure information, abstracted the information on anatomic location, stage, and histological type of the cancer. Colorectal cancer was defined according to the International Classification of Diseases, Ninth Revision (ICD-9) (Puckett, 1986). During the follow-up period (1980–2008), a total of 1601 incident colorectal cancer cases were documented.

### Measurement of plasma C-peptide levels

Detailed information on blood draw, transportation, and storage in the NHS is reported in detail elsewhere (Hankinson *et al*, 1995). In brief, blood samples were collected from 32 826 NHS participants between 1989 and 1990. We used plasma C-peptide data available among 1862 control participants in case–control studies of C-peptide and diabetes, breast cancer, hypertension, colorectal polyps, and colorectal cancer. We excluded participants with diabetes and a previous cancer diagnosis (except non-melanoma skin cancer) before blood draw to ensure that C-peptide levels were not influenced by these diseases. We also restricted the analysis to the women who have been fasting at least 9 h at the time of blood draw. As previously described (Wu *et al*, 2004), fasting levels of plasma C-peptide were measured by enzyme-linked immunosorbent assay and a radioimmunoassay method with all the CV <11% using blinded quality control samples.

### Assessment of magnesium intake

A validated semi-quantitative food frequency questionnaire (FFQ) (Willett, 1998) was first administered in 1980 to collect information on usual dietary intake over the past year and then in 1984, 1986, and every 4 years thereafter. Nine possible frequency choices were available, ranging from ‘almost never’ to ‘6 or more times per day’. Nutrient intakes were calculated by multiplying the frequency of each food consumed and the nutrient content of specified portion sizes. The food composition values for magnesium were mainly derived from the US Department of Agriculture, Agricultural Research Service (1998). In addition to foods, magnesium intake from supplemental sources was derived using information collected on multivitamin use and specific magnesium supplements. Total intake of magnesium was calculated by summing up the amounts from both food and supplemental sources. The nutrients including the total and dietary magnesium intakes were adjusted for total energy intake using the residual method (Willett, 1998). Although total magnesium intake was not evaluated in the validation study, we have conducted validation studies on major contributors of total magnesium (Willett *et al*, 1985; Salvini *et al*, 1989). The major contributors to (overall contributes to ∼60–70%) total magnesium intake in our cohort included the cold breakfast cereal, skim milk, coffee, multivitamins, dark bread, nuts, spinach, broccoli, and banana. The Pearson correlation coefficients between the mean intakes from the four 1-week dietary records and those from the questionnaires completed after the dietary records ranged from 0.69 for broccoli to 0.81 for skim milk after accounting for random errors in dietary records (Salvini *et al*, 1989). In addition, the same FFQ (essentially after 1980 in NHS) was used in a cohort of male health professionals, the Pearson correlation coefficient for total magnesium between the average of two 1-week dietary records and the FFQ completed after the dietary records were 0.71 after accounting for random errors in dietary records (Rimm *et al*, 1992).

### Assessment of other variables

Information on other dietary factors such as consumption of red meat, processed meat, alcohol, folate, calcium, vitamin D, fruits, and vegetables was also collected from the baseline and subsequent FFQs. In addition, we inquired about potential colorectal cancer risk factors such as height, body weight, physical activity, cigarette smoking, family history of colorectal cancer, aspirin use, menopausal status, and postmenopausal hormone use in the biennial questionnaires.

### Statistical analyses

We calculated person-time for each participant from the date of baseline questionnaire return to the date of death, colorectal cancer diagnosis, or the end of follow-up (1 June 2008), whichever came first. We used a Cox proportional hazards regression model (Cox, 1972) to calculate RRs and 95% confidence intervals (CIs) and adjusted simultaneously for age (in months) and year of questionnaire return. We observed no violation of the proportional hazard assumption based on the likelihood ratio test that compared the model with and without the interaction terms between magnesium intake and age or follow-up time.

To take advantage of the large number of cases and to examine relative extreme intake categories, we categorised energy-adjusted magnesium intakes into deciles based on the distribution in the study population. We assigned the median values of these categories and entered these values as continuous variables into the model to conduct trend tests. In addition to adjusting for age, in the multivariate models, we further adjusted for established non-dietary risk factors (models 2 and 3) and additional dietary factors (model 3 only, see Table 2 footnote for the list of these variables and their categorisations). We further conducted additional analyses in which we adjusted for multivitamin use, total intakes of zinc, vitamin B6, vitamin B12, fibre, retinol, *β*-carotene, potassium, vitamin E, saturated fat, phosphorous, and glycemic load. Further adjustment of these factors, one at a time or simultaneously, did not materially alter the results and thus were not included in the final model. We modelled all covariates as time-varying variables to take into account potential changes over follow-up. To represent better long-term dietary intake (Hu *et al*, 1999) and to minimise the impact of random measurement errors using dietary assessments, we calculated the cumulative average intakes of total and dietary magnesium intake. In addition, we analysed magnesium from supplemental sources using tertile categories because intake of supplemental magnesium was low in this study population (contributing to <8% of total magnesium).

To examine the interaction between magnesium and calcium intakes observed in one case–control study (Dai *et al*, 2007) of colorectal adenomas and hyperplastic polyps, we first tested whether associations between total magnesium intake (in quintiles) and colorectal cancer risk varied by total calcium intake (<854, ⩾854 mg per day; median). Second, we examined whether the associations with total magnesium intake varied by the Ca:Mg ratio (<3.0, ⩾3.0; median). Further, given that magnesium might influence colorectal cancer risk by improving insulin sensitivity, associations with magnesium intake might be stronger among overweight/obese individuals with possible insulin resistance, as suggested in one prior study (van den Brandt *et al*, 2007). We thus evaluated whether the associations with magnesium intake varied by body mass index (BMI) (<25, ⩾25 kg m^–2^). Finally, we examined whether associations with magnesium intake varied by alcohol consumption (non-drinkers, >0–<10, ⩾10 g per day), vitamin D intake (<298, ⩾298 IU per day), and physical activity (<6, ⩾6 MET-hours per week). We used a Wald test to examine whether the *β*-coefficients of the cross-product terms between these variables and total magnesium intake were statistically significant.

Regarding the C-peptide analysis, we log-transformed concentrations of C-peptide because the distribution was skewed. We used multivariable (MV) linear regression with robust variance (PROC MIXED with empirical statement) (White, 1980) to investigate the association between magnesium intakes and plasma concentrations of C-peptide. We adjusted for the same non-dietary lifestyle and dietary factors (measured in 1990, if applicable) as the ones listed in the full-cohort analysis.

All statistical analyses were two-sided and a *P*-value <0.05 was considered statistically significant. We conducted all analyses using the SAS software (Version 9.2, SAS Institute, Inc., Cary, NC, USA).

## Results

A total of 1601 incident colorectal cancer cases were documented among 85 924 women during 2 238 710 persons-years from 1980 to 2008. Magnesium intake increases over time in our study population. For example, the mean intake of total magnesium increased from 287 mg per day in 1984 and 300 mg per day in 1990 to 377 mg per day in 2008. Dietary sources accounted for the majority of total magnesium intake and averaged over the entire follow-up, magnesium intake from supplemental sources contributed ∼8% of total magnesium intake.

Selected demographic and lifestyle characteristics and potential confounding factors were compared across the intake levels of total and dietary magnesium (Table 1). Women with higher total and dietary magnesium intakes were generally comparable with women with lower intakes with respect to BMI, family history of colorectal cancer, and total calorie intake. However, women with higher magnesium intakes were slightly older, more likely to be physically active and use aspirin and multivitamin as well as consume fruits and vegetables, dietary fibre, total calcium, total vitamin D, and total folate, but less likely to smoke and consume alcohol, red meat, and processed meat.

Total magnesium intake was significantly associated with a lower risk of colorectal cancer risk in age-adjusted model but the association was attenuated after adjustment for lifestyle factors and became largely non-significant after further adjustment for other dietary factors (Table 2). The attenuation was mainly driven by adjustment for total calcium, total vitamin D, and total folate intake. Of note, significant inverse associations with total calcium and vitamin D intake persisted in the same MV models, suggesting that magnesium has a weaker relation with risk of colorectal cancer compared with calcium and vitamin D. Compared with the first decile of total magnesium intake (D1, <229 mg per day), the MV-adjusted RRs ranged from 0.78 to 0.86 in the third to seventh deciles (253–328 mg per day), and ranged from 0.91 to 1.03 in the top three deciles (329–1110 mg per day). For colon cancer, women in most of the categories above the bottom decile were at 10–20% lower risk from deciles 2 to 10. Magnesium from supplemental sources was not significantly associated with colorectal cancer risk in either age-adjusted or MV-adjusted models (data not shown). We observed an overall non-significant association between total magnesium intake and risk of proximal or distal colon cancer (data not shown). For example, compared with the first decile of total magnesium intake, the MV RRs for distal colon cancer ranged from 0.56 to 1.02 for deciles 2–10 (*P* for trend=0.50) with the only significant RR observed in decile 4 (RR=0.56, 95% CI: 0.36–0.87). For the same comparison, the MV RRs for proximal colon cancer ranged from 0.66 to 1.04 for deciles 2–10 (*P* for trend=0.26) with the lowest RR observed in decile 6 (RR=0.66, 95% CI: 0.46–0.96). In addition, generally non-significant associations were seen when we modelled magnesium intake as quintiles (data not shown).

Overall, no significant interactions were observed between total magnesium and calcium intakes (Table 3). In addition, no clear pattern was observed when we examined associations with total magnesium and calcium intake in relation to colorectal cancer risk by Ca:Mg in our study (data not shown). Moreover, the associations were not significantly modified by BMI (<25, ⩾25 kg m^–2^), alcohol consumption (non-drinkers, >0–<10, ⩾10 g per day), vitamin D intake (<298, ⩾298 IU per day), and physical activity (<6, ⩾6 MET-hours per week), or menopausal status (premenopausal, postmenopausal) (data not shown). Of note, similar results were observed for colorectal cancer when we categorised the BMI into three groups (<25, 25–30, ⩾ 30 kg m^–2^) and sensitivity analysis of excluding diabetic women or restricting to older women (i.e., ⩾65 years) yielded similar null results (data not shown). Because ∼70–80% of colorectal cancer in developed countries is colon cancer (Wei *et al*, 2004), we classified 31 colorectal cancer cases with unknown information on subsite into the colon cancer. Sensitivity analysis of excluding these cases did not change the results. Results for dietary magnesium intake were similar to those observed for total magnesium intake (data not shown). In addition, similar non-significant associations were seen when we analysed baseline magnesium intake (data not shown).

We tested whether magnesium intake was associated with plasma C-peptide levels among 1862 cohort members with fasting blood samples, who were free of cancer and diabetes at the blood draw. In the age-adjusted model, higher intakes of magnesium were associated with lower fasting mean plasma C-peptide levels (*P* for trend=0.002) (Table 4). However, this association did not persist after further adjustment for non-dietary lifestyle and dietary factors (*P* for trend=0.61). The non-significant association was not varied (all *P* for trend >0.52) when the analysis was stratified by BMI (<25, 25–30, ⩾ 30 kg m^–2^) (data not shown).

## Discussion

In this large prospective study of middle-aged to older American women, we observed no significant association between magnesium intakes with risk of colorectal cancer. In addition, we found no significant modification by BMI, alcohol consumption, calcium intake, vitamin D intake, physical activity, and menopausal status. Moreover, magnesium intake was not significantly independently associated with fasting plasma concentrations of C-peptide, a marker for insulin secretion.

The current epidemiologic data regarding the association between magnesium intake and colorectal cancer incidence have been mixed. Among the six studies (Larsson *et al*, 2005; Folsom and Hong, 2006; Lin *et al*, 2006; van den Brandt *et al*, 2007; Ma *et al*, 2010; Li *et al*, 2011), an inverse association was first suggested in a cohort of Swedish women for both colon (⩾255 *vs* <209, RR=0.66, 95% CI: 0.41–1.07, *P* for trend=0.08) and rectal cancer (same comparison, RR=0.45, 95% CI: 0.22–0.89, *P* for trend=0.02) (Larsson *et al*, 2005). The Iowa Women's Health Study subsequently reported that total magnesium intake was associated with a lower risk of colon cancer (extreme quintile comparison, >351 *vs* <245 mg per day, RR=0.77, 95% CI: 0.58–1.03, *P* for trend=0.04) but not with rectal cancer (same comparison, RR=0.95, 95% CI: 0.56–1.60, *P* for trend=0.96) (Folsom and Hong, 2006). No associations were observed in the Women's Health Study (extreme quintile comparison, >392 *vs* <279 mg per day, RR=0.97, 95% CI: 0.63–1.49, *P* for trend=0.88) (Lin *et al*, 2006), the Netherlands Cohort Study (extreme quintile comparison, >373 *vs* <286 mg per day, RR=0.91, 95% CI: 0.62–1.35, *P* for trend=0.50 for men; >326 *vs* <256, RR=0.89, 95% CI: 0.59–1.35, *P* for trend=0.77 for women ) (van den Brandt *et al*, 2007), and a cohort study conducted in Germany (⩾381 *vs* <261 mg per day, RR=1.27, 95% CI: 0.76–2.10, *P* for trend=0.41) (Li *et al*, 2011). In the only study conducted in an Asian population, a significant inverse association between dietary magnesium intake and colorectal cancer risk was observed in Japanese men (⩾327 *vs* <238 mg per day, RR=0.65, 95% CI: 0.40–1.03, *P* for trend=0.04) but not in women (⩾316 *vs* <237 mg per day, RR=1.15, 95% CI: 0.60–2.21, *P* for trend=0.69) (Ma *et al*, 2010). Of note, among these cohorts, the intake level and the inter-quintile intake range was the lowest in the Swedish study (Larsson *et al*, 2005) (209–255 mg per day) compared with all other studies (minimum inter-quintile intake of 245 and maximum of 392 mg per day). The inverse associations observed in the Swedish study (Larsson *et al*, 2005) suggest that individual's colorectal risk may be increased only at very low magnesium intake. In support of this, Ma *et al* (2006) reported that magnesium intake up to 325 mg per day was associated with insulin sensitivity but provide no further benefit above this level.

The efficacy and safety of magnesium from supplements on colorectal cancer risk is unclear although some animal data showed that magnesium supplementation may decrease the occurrence of experimentally induced colon cancer by potentially binding to potentially carcinogenic bile acids (Mori *et al*, 1993). Current human data on magnesium supplements are sparse and low prevalence of magnesium supplementation use (i.e., <10%) in our study and the earlier studies (Folsom and Hong, 2006; Lin *et al*, 2006; van den Brandt *et al*, 2007) limited our ability to draw firm conclusions regarding magnesium supplement use and colorectal cancer risk.

A recent case–control study indicated a possible interaction between calcium and magnesium intake in relation to risk of colorectal adenoma and hyperplastic polyps (Dai *et al*, 2007). In that study, a significant inverse association was observed with magnesium intake only among the participants with low calcium to magnesium ratio (Dai *et al*, 2007), supporting their hypothesis that a low Ca:Mg intake ratio may promote magnesium transportation. We evaluated this hypothesis and found no overall clear pattern. Nonetheless, the influence of increasing Ca:Mg intake ratio on health is unclear. A high Ca:Mg intake ratio, possibly caused by low dietary magnesium intake and a steady increase in calcium intake, might link to insulin resistance, hyperinsulinemia, type 2 diabetes, and cardiovascular diseases (Rosanoff, 2010). The adequacy of calcium and magnesium and their balance for optimal health warrant future investigation.

Magnesium as a cofactor for several enzymes for glucose metabolism and the inverse association reported between magnesium intake and risk of type 2 diabetes in a meta-analysis of seven cohort studies (Larsson and Wolk, 2007) supports a potential role of magnesium in glucose homeostasis and insulin action. In addition, higher magnesium intake has been shown to be associated with lower fasting insulin concentrations among women without diabetes (Fung *et al*, 2003). We observed a significant inverse association between magnesium intake and levels of plasma C-peptide, a marker of insulin secretion (Bonser and Garcia-Webb, 1984), but results were significantly attenuated in MV-adjusted model. In addition, individuals with insulin resistance might benefit from magnesium intake but observational studies have shown mixed results. In contrast to the significant inverse trends between magnesium intake and colorectal cancer risk observed in overweight individuals in the study conducted in the Netherlands (van den Brandt *et al*, 2007), we found no clear indication of this interaction between magnesium intake and BMI, which was consistent with the results from three other studies (Folsom and Hong, 2006; Lin *et al*, 2006; Ma *et al*, 2010).

Several limitations of our study merit consideration. Measurement error associated with assessing magnesium intake exists. In contrast to using a single baseline measurement of magnesium intake in the majority of early studies, the comprehensive updated measurements of magnesium intake used in the current study allowed us not only to reduce measurement error but also take into account changes in intake over time. In addition, results from multivariate models in which we controlled for non-dietary and other dietary factors need to be interpreted with caution because magnesium intake is correlated with intake of calcium (*r*=0.5) and other nutrients. As with earlier studies (Folsom and Hong, 2006; Lin *et al*, 2006; van den Brandt *et al*, 2007), we had limited power to evaluate the association with magnesium from supplemental sources. Although the overall study is large, we had limited power to examine potential interactions, especially with calcium.

Major strengths of our study included its large size, prospective design, repeated measurements, long follow-up time, and high follow-up rate. In addition, the fasting pre-diagnostic plasma C-peptide levels among non-diabetic individuals are more likely to represent the long-term insulin levels because the levels are less likely influenced by disease status.

In summary, although our study among middle-aged US women did not support an overall independent and linear association of magnesium intake with colorectal cancer risk, we cannot rule out a possible non-linear relationship, especially for colon cancer and among individuals with low calcium intake. A meta-analysis and studies of genetic variants of the magnesium metabolising pathway might be informative.

## Figures and Tables

**Table 1:** Age-standardised characteristics (in 1990) by quintile (Q) of total and dietary magnesium intake in the Nurses’ Health Study

	**Total magnesium intake**			**Dietary magnesium intake**		
**Characteristics**	**Q1**	**Q3**	**Q5**	**Q1**	**Q3**	**Q5**
Intake of magnesium, range (median) (mg per day)	<251.5 (229.0)	283.1–312.0 (297.4)	>348.9 (379.7)	<244.8 (224.5)	272.5–297.0 (285.0)	>329.3 (355.2)
Age, mean (s.d.), years	54.8 (7.1)	56.6 (7.1)	58.5 (6.9)	54.7 (7.2)	56.7 (7.1)	58.8 (6.8)
Body mass index (kg m^–2^), mean (s.d.)^a^	25.3 (5.0)	25.1 (4.4)	24.7 (4.2)	25.3 (4.9)	25.0 (4.4)	24.6 (4.1)
Physical activity (MET-hours per week), mean (s.d.)^b^	10.9 (14.2)	14.4 (17.3)	20.0 (23.0)	10.7 (14.0)	14.4 (16.7)	20.2 (23.5)
History of colorectal cancer in a parent or sibling (%)	11	11	11	11	11	11
Current smokers (%)	19	16	15	22	16	14
Regular aspirin use (%)^c^	38	40	49	39	41	40
Multivitamin use (%)	24	36	50	31	38	46
						
*Dietary intake, mean (s.d.)*						
Total energy (kcal per day)	1683 (431)	1716 (420)	1678 (423)	1705 (437)	1712 (420)	1682 (417)
Total carbohydrate (g per day)	194 (33)	198 (31)	210 (32)	192 (34)	197 (30)	212 (32)
Total fat (g per day)	60.0 (10.4)	56.2 (9.7)	50.6 (10.3)	60.4 (10.5)	56.3 (9.6)	49.8 (10.0)
Total protein (g per day)	69.9 (12.6)	76.3 (12.3)	81.5 (14.8)	69.1 (12.5)	76.4 (12.0)	82.2 (14.6)
Fruits and vegetables (servings per day)	3.8 (1.4)	5.1 (1.7)	6.4 (2.3)	3.8 (1.5)	5.1 (1.7)	6.4 (2.3)
Dietary fibre (g per day)^d^	13.9 (2.8)	16.9 (3.4)	21.0 (5.0)	13.6 (2.7)	16.8 (3.1)	21.6 (4.9)
Alcohol consumption (g per day)	6.1 (10.6)	6.4 (9.0)	5.7 (8.2)	6.7 (11.2)	6.4 (9.2)	5.4 (7.9)
Total calcium intake (mg per day)^d^	703 (243)	898 (268)	1151 (338)	723 (253)	909 (282)	1112 (334)
Total vitamin D intake (IU per day)^d^	232 (143)	316 (165)	461 (219)	254 (161)	324 (177)	422 (210)
Total folate intake (*μ*g per day)^d^	297 (134)	380 (146)	526 (195)	317 (150)	389 (156)	490 (187)
Beef, pork, or lamb as a main dish (servings per week)	2.5 (1.4)	2.1 (1.2)	1.6 (1.1)	2.4 (1.3)	2.1 (1.2)	1.6 (1.0)
Processed meat intake (servings per week)	1.3 (1.4)	1.0 (1.1)	0.6 (0.8)	1.3 (1.4)	1.0 (1.0)	0.6 (0.8)

a Body mass index was calculated as weight in kilograms divided by the square of height in metres.

b MET denotes metabolic equivalent. MET-hours=sum of the average time per week spent in each activity × MET value of each activity.

c Regular aspirin user was defined as consumption of two or more 325 mg tablets per week. Non-regular user was defined otherwise.

d Nutrient values were energy-adjusted intake.

**Table 2:** Relative risks (RRs, 95% CIs) for incident colorectal cancer by deciles of total magnesium intake in the Nurses’ Health Study (1980–2008)

	**Deciles of total magnesium intake: median (range, mg per day)**										
	**D1: 211 (<230)**	**D2: 242 (230–251)**	**D3: 260 (252–268)**	**D4: 275 (269–282)**	**D5: 289 (283–297)**	**D6: 304 (298–311)**	**D7: 318 (312–328)**	**D8: 336 (329–347)**	**D9: 359 (348–376)**	**D10: 401 (>376)**	***P* for trend**
*Colorectal cancer (*n=*1601)*											
No. of cases	165	169	155	144	149	161	154	164	181	159	
Person-years	216 769	222 057	225 281	223 824	226 180	223 364	225 936	224 011	226 164	225 126	
Model 1^a^	1.0 (Reference)	0.93 (0.75, 1.15)	0.81 (0.65, 1.01)	0.72 (0.58, 0.91)	0.74 (0.59, 0.92)	0.77 (0.62, 0.95)	0.72 (0.57, 0.89)	0.75 (0.60, 0.93)	0.80 (0.64, 0.98)	0.67 (0.53, 0.83)	<0.001
Model 2^b^	1.0 (Reference)	0.97 (0.78, 1.20)	0.86 (0.69, 1.07)	0.78 (0.62, 0.97)	0.80 (0.64, 1.00)	0.84 (0.67, 1.05)	0.81 (0.64, 1.01)	0.85 (0.68, 1.06)	0.92 (0.74, 1.14)	0.78 (0.63, 0.98)	0.09
Model 3^c^	1.0 (Reference)	0.96 (0.77, 1.19)	0.85 (0.68, 1.07)	0.78 (0.61, 0.98)	0.82 (0.64, 1.03)	0.86 (0.68, 1.10)	0.84 (0.66, 1.08)	0.91 (0.72, 1.16)	1.02 (0.81, 1.31)	0.93 (0.73, 1.22)	0.77
											
*Colon cancer (*n=*1252)*											
No. of cases	132	127	123	113	111	128	123	128	150	117	
Person-years	216 799	222 088	225 307	223 847	226 211	223 394	225 963	224 046	226 191	225 165	
Model 1^a^	1.0 (Reference)	0.86 (0.68, 1.10)	0.79 (0.62, 1.02)	0.70 (0.54, 0.90)	0.68 (0.53, 0.87)	0.75 (0.59, 0.96)	0.70 (0.55, 0.90)	0.71 (0.56, 0.91)	0.81 (0.64, 1.02)	0.60 (0.46, 0.77)	<0.001
Model 2^b^	1.0 (Reference)	0.90 (0.70, 1.15)	0.84 (0.66, 1.08)	0.74 (0.58, 0.96)	0.73 (0.57, 0.95)	0.82 (0.64, 1.05)	0.79 (0.61, 1.01)	0.80 (0.62, 1.03)	0.93 (0.73, 1.18)	0.70 (0.54, 0.90)	0.06
Model 3^c^	1.0 (Reference)	0.89 (0.69, 1.14)	0.83 (0.64, 1.07)	0.74 (0.57, 0.96)	0.74 (0.56, 0.97)	0.83 (0.64, 1.09)	0.81 (0.62, 1.07)	0.84 (0.64, 1.11)	1.01 (0.77, 1.33)	0.81 (0.61, 1.10)	0.77
											
*Rectal cancer (*n=*349)*											
No. of cases	33	42	32	31	38	33	31	36	31	42	
Person-years	216 881	222 161	225 392	223 929	226 277	223 469	226 048	224 115	226 294	225 227	
Model 1^a^	1.0 (Reference)	1.19 (0.75, 1.89)	0.86 (0.53, 1.41)	0.83 (0.51, 1.36)	0.98 (0.61, 1.57)	0.83 (0.51, 1.34)	0.77 (0.47, 1.26)	0.90 (0.56, 1.45)	0.73 (0.45, 1.20)	0.96 (0.61, 1.53)	0.31
Model 2^b^	1.0 (Reference)	1.26 (0.79, 1.99)	0.93 (0.57, 1.52)	0.91 (0.56, 1.50)	1.09 (0.68, 1.75)	0.92 (0.57, 1.50)	0.88 (0.54, 1.45)	1.05 (0.65, 1.70)	0.86 (0.52, 1.42)	1.17 (0.73, 1.86)	0.97
Model 3^c^	1.0 (Reference)	1.26 (0.78, 1.98)	0.94 (0.57, 1.55)	0.93 (0.55, 1.54)	1.14 (0.69, 1.87)	0.99 (0.58, 1.66)	0.97 (0.57, 1.65)	1.19 (0.71, 2.03)	1.04 (0.60, 1.82)	1.54 (0.90, 2.66)	0.23

Abbreviations: CI=confidence interval; MET=metabolic equivalent.

a Model 1 was adjusted for age (in months).

b Model 2 was adjusted for age (in months), smoking before age 30 (0, 1–4, 5–10, or >10 pack-years), history of colorectal cancer in a parent or sibling (yes, no), history of endoscopy (yes, no), regular aspirin use (yes, no), body mass index (<25, 25–<30, ⩾30 kg m^–2^), physical activity (<3, 3–<27, ⩾27 MET-hours per week), postmenopausal hormone use (premenopausal, never, past, or current user).

c Model 3 was further adjusted for consumption of processed meat (quintiles), consumption of beef, pork, or lamb as a main dish (quintiles), alcohol consumption (0–<5, 5–<10, 10–<15, or ⩾15 g per day), energy-adjusted total calcium intake (quintiles), total folate (quintiles), total vitamin D intake (quintiles), and total energy intake (quintiles).

**Table 3:** Multivariable^a^ relative risks (RRs, 95% CIs) for the joint classification of total magnesium intake and total calcium and body mass index in the Nurses’ Health Study (1980–2008)

	**Quintiles of total magnesium intake (mg per day), median (range)**										
	**229.0 (79–251.5)**		**268.3 (251.5–283.0)**		**297.4 (283.1–312.0)**		**328.5 (312.1–348.9)**		**379.7 (349.0–1100.0)**		
	**No. cases**	**RR (95% CI)**	**No. cases**	**RR (95% CI)**	**No. cases**	**RR (95% CI)**	**No. cases**	**RR (95% CI)**	**No. cases**	**RR (95% CI)**	***P*_interaction_**
*Colorectal cancer (*n=*1601)*											
Total calcium intake											0.17
<Median^b^	174	1.0 (Reference)	198	0.84 (0.69, 1.01)	142	0.76 (0.61, 0.94)	110	0.79 (0.63, 1.00)	77	0.98 (0.75, 1.29)	
⩾Median^b^	60	0.76 (0.57, 1.02)	101	0.66 (0.52, 0.84)	168	0.79 (0.64, 0.98)	208	0.80 (0.65, 0.98)	263	0.85 (0.69, 1.05)	
BMI (kg m^–2^)											0.75
<25	152	1.0 (Reference)	144	0.88 (0.69, 1.10)	169	1.02 (0.81, 1.28)	161	0.97 (0.76, 1.23)	171	1.04 (0.81, 1.33)	
⩾25	182	1.29 (1.04, 1.61)	155	1.03 (0.82, 1.29)	141	0.94 (0.74, 1.19)	157	1.08 (0.85, 1.38)	169	1.29 (1.01, 1.64)	
											
*Colon cancer (*n=*1252)*											
Total calcium intake											0.35
<Median^b^	208	1.0 (Reference)	156	0.87 (0.70, 1.07)	109	0.77 (0.60, 0.98)	87	0.81 (0.63, 1.06)	59	0.97 (0.72, 1.33)	
⩾Median^b^	51	0.84 (0.61, 1.15)	80	0.66 (0.51, 0.87)	130	0.78 (0.61, 0.99)	164	0.80 (0.63, 1.01)	208	0.85 (0.67, 1.08)	
BMI (kg m^–2^)											0.78
<25	120	1.0 (Reference)	111	0.84 (0.64, 1.09)	131	0.98 (0.76, 1.27)	125	0.91 (0.70, 1.19)	134	0.98 (0.74, 1.30)	
⩾25	139	1.22 (0.96, 1.57)	125	1.02 (0.79, 1.32)	108	0.88 (0.67, 1.15)	126	1.06 (0.81, 1.38)	133	1.23 (0.93, 1.62)	
											
*Rectal cancer (*n=*349)*											
Total calcium intake											0.23
<Median^b^	66	1.0 (Reference)	33	0.74 (0.50, 1.10)	38	0.74 (0.48, 1.14)	36	0.72 (0.44, 1.19)	37	1.03 (0.59, 1.80)	
⩾Median^b^	9	0.51 (0.25, 1.03)	30	0.64 (0.38, 1.07)	33	0.82 (0.53, 1.27)	31	0.81 (0.52, 1.25)	36	0.85 (0.55, 1.32)	
BMI (kg m^–2^)											0.19
<25	32	1.0 (Reference)	42	1.04 (0.63, 1.71)	33	1.19 (0.73, 1.94)	23	1.20 (0.72, 2.00)	18	1.29 (0.75, 2.19)	
⩾25	43	1.59 (1.00, 2.53)	21	1.05 (0.63, 1.74)	38	1.17 (0.71, 1.96)	44	1.19 (0.70, 2.01)	55	1.55 (0.91, 2.65)	

Abbreviations: CI=confidence interval; BMI=body mass index; MET=metabolic equivalent.

a Adjusted for age (in months), smoking before age 30 (0, 1–4, 5–10, or >10 pack-years), history of colorectal cancer in a parent or sibling (yes, no), history of endoscopy (yes, no), regular aspirin use (yes, no), BMI (<25, 25–<30, ⩾30 kg m^–2^, not for analysis of BMI), physical activity (<3, 3–<27, ⩾27 MET-hours per week), postmenopausal hormone use (premenopausal, never, past, or current user), consumption of processed meat (quintiles), consumption of beef, pork, or lamb as a main dish (quintiles), alcohol consumption (0–<5, 5–<10, 10–<15, or ⩾15 g per day), energy-adjusted total calcium intake (quintiles; not for analysis of calcium), total folate (quintiles), total vitamin D intake (quintiles), and total energy intake (quintiles).

b Median value 854 mg per day was used.

**Table 4:** Geometric means (s.d.) for fasting C-peptide (ng ml^–1^) for 1862 women by deciles (D) of magnesium intake in the Nurses’ Health Study

	**Magnesium deciles**										
**Intakes**	**D1**	**D2**	**D3**	**D4**	**D5**	**D6**	**D7**	**D8**	**D9**	**D10**	***P* for trend**
*Total magnesium*^a^ *(mg per day)*											
Medians	211	240	258	272	287	304	322	344	373	434	
No. of controls	197	187	188	184	180	187	186	173	199	183	
Model 1^b^	1.51 (1.38, 1.66)	1.46 (1.34, 1.58)	1.34 (1.22, 1.47)	1.33 (1.21, 1.48)	1.41 (1.28, 1.56)	1.35 (1.24, 1.47)	1.41 (1.29, 1.53)	1.35 (1.24, 1.48)	1.30 (1.21, 1.41)	1.26 (1.17, 1.36)	0.002
Model 2^c^	1.65 (1.50, 1.81)	1.63 (1.50, 1.78)	1.41 (1.28, 1.55)	1.53 (1.38, 1.70)	1.58 (1.43, 1.74)	1.54 (1.41, 1.69)	1.62 (1.47, 1.77)	1.57 (1.43, 1.72)	1.53 (1.41, 1.67)	1.52 (1.40, 1.66)	0.39
Model 3^d^	1.61 (1.45, 1.78)	1.59 (1.45, 1.74)	1.40 (1.27, 1.55)	1.49 (1.33, 1.67)	1.55 (1.41, 1.71)	1.51 (1.38, 1.66)	1.57 (1.42, 1.73)	1.57 (1.42, 1.73)	1.51 (1.38, 1.65)	1.50 (1.36, 1.66)	0.61
											
*Dietary magnesium*^a^ *(mg per day)*											
Medians	208	237	252	2656	278	294	308	326	347	387	
No. of controls	198	183	190	177	201	193	171	188	174	189	
Model 1^b^	1.52 (1.40, 1.65)	1.42 (1.29, 1.55)	1.44 (1.33, 1.56)	1.33 (1.20, 1.48)	1.41 (1.30, 1.53)	1.37 (1.25, 1.49)	1.29 (1.18, 1.41)	1.41 (1.30, 1.53)	1.26 (1.15, 1.37)	1.27 (1.18, 1.38)	0.0007
Model 2^c^	1.66 (1.53, 1.80)	1.60 (1.45, 1.76)	1.54 (1.41, 1.68)	1.46 (1.31, 1.63)	1.62 (1.49, 1.76)	1.56 (1.42, 1.71)	1.48 (1.35, 1.62)	1.63 (1.49, 1.79)	1.47 (1.34, 1.61)	1.54 (1.41, 1.68)	0.21
Model 3^d^	1.63 (1.49, 1.79)	1.56 (1.41, 1.72)	1.52 (1.39, 1.68)	1.44 (1.29, 1.61)	1.59 (1.46, 1.74)	1.53 (1.39, 1.70)	1.43 (1.30, 1.58)	1.58 (1.43, 1.75)	1.44 (1.31, 1.60)	1.50 (1.36, 1.66)	0.24

Abbreviation: MET=metabolic equivalent.

a The magnesium intakes were based on 1990 data from the Nurses’ Health Study. Geometric mean values were centred at age 60 years.

b Model 1 was adjusted for age at blood draw (in months).

c Model 2 was adjusted for age at blood draw (in months), smoking before age 30 (0, 1–4, 5–10, or >10 pack-years), history of colorectal cancer in a parent or sibling (yes, no), history of endoscopy (yes, no), regular aspirin use (yes, no), body mass index (<25, 25–<30, ⩾30 kg m^–2^), physical activity (<3, 3–<27, ⩾27 MET-hours per week), postmenopausal hormone use (premenopausal, never, past, or current user).

d Model 3 was further adjusted for consumption of processed meat (quintiles), consumption of beef, pork, or lamb as a main dish (quintiles), alcohol consumption (0–<5, 5–<10, 10–<15, or ⩾15 g per day), energy-adjusted total calcium intake (quintiles), total folate (quintiles), total vitamin D intake (quintiles), and total energy intake (quintiles).
